# Effects of different irrigation methods on the spatial and temporal distribution of soil temperature

**DOI:** 10.1371/journal.pone.0332649

**Published:** 2025-09-18

**Authors:** Hong Chen, Wenxin Liu, Guodong Wang, Yumeng Zheng, Xueting Fan, Licun Zhang, Fei Liang

**Affiliations:** 1 School of Resources and Environment, Yili Normal University, Yining, China; 2 Institute of Farmland Water Conservancy and Soil-fertilizer, Xinjiang Academy of Agricultural Reclamation Science, Shihezi, China; 3 Institute of Resources and Ecology, Yili Normal University, Yining, China; Ardakan University, IRAN, ISLAMIC REPUBLIC OF

## Abstract

Drip irrigation delivers water uniformly and precisely to the root zone, thereby influencing the soil hydrothermal regime of the soil. While this characteristic makes drip irrigation a promising water-saving technique, there are limited long-term comparative studies on drip irrigation and flood irrigation, which cannot comprehensively and systematically elucidate the impact of water-saving irrigation on soil temperature in Northwest China. We conducted a five-year field experiment from 2017 to 2021, focusing on the comprehensive impact of two irrigation methods (drip irrigation and flood irrigation) on soil temperature at different depths and at different positions (dripper source, root zone, inter-row buffer) during the maize growth periods. The results revealed that soil temperature and meteorological temperature had an extremely significant correlation (p < 0.001), and that the soil surface layer was more susceptible to air temperature influences and was positively correlated with atmospheric temperature. Compared to flood irrigation, drip irrigation increased soil temperature by 1.4%, while soil temperature exhibited a significant negative correlation with soil depth. Specifically, at 0 cm depth, the soil temperature varied most significantly, ranging from 19.79°C to 28.38°C, while at 90 cm, it was 19.98°C – 21°C. Compared to other positions, root zone soil temperature increased by 0.5% to 2.25%. Therefore, drip irrigation not only enhances soil temperature but also optimizes hydrothermal conditions for crop roots. The contribution of this study lies in its integration of multi – year, multi – depth, and multi – positional monitoring under field conditions, which fills a major gap in understanding how irrigation systems influence soil thermal regimes in dry locations. This study provides an actionable theoretical framework for optimizing water use efficiency and crop productivity in water-scarce agroecosystems, while also laying a solid scientific foundation for the development of sustainable management models for agricultural water resources in arid regions.

## 1. Introduction

Soil temperature is a critical environmental factor governing multiple soil functions. [1]. An appropriate soil temperature not only accelerates the evaporation of soil moisture and enhances the water absorption rate of crop roots [2], but also enhances the activity of soil microorganisms and crop roots, accelerates the release and transformation of soil nutrients, and promotes crop growth and development [3]. Soil is a highly heterogeneous medium with pronounced spatial and temporal variations in its physical, chemical, and biological properties [4,5]. These heterogeneities can modulate soil temperature and moisture responses to environmental drivers (e.g., precipitation, solar radiation, irrigation) by altering heat and water transport pathways. Therefore, in numerous instances crop growth and yield are more significantly influenced by soil temperature than by ambient air temperature [6,7]. Empirical evidence suggests that many factors exert an influence on soil temperature, including meteorological conditions, topographical features, ground cover, soil moisture content, and vegetation type [8,9]. Among them, soil moisture content is a significant factor affecting soil temperature [10,11].

However, irrigation methods are one of the important measures affecting soil moisture content. Different irrigation methods can directly affect the distribution of water in the soil [12,13]. Drip irrigation facilitates water infiltration from the emitter to the root zone and subsequently to the inter-row area, whereas flood irrigation exhibits the opposite pattern, showing a law of infiltration from the wide row to the root and then to the narrow row. Drip irrigation preferentially supplements the dripper and soil root zone, while flood irrigation preferentially supplements the soil moisture in the wide row [14,15]. At present, with the rapid development of drip irrigation in Xinjiang, the irrigation methods have turned from traditional irrigation to efficient water-saving drip irrigation [16,17]. The area of under-membrane drip irrigation has exceeded 70 million acres [18]. Previous studies have shown that drip irrigation takes advantage of the benefits of drip irrigation and film mulching, has significant advantages in saving water and improving yields, and creates suitable conditions for crop growth in arable soils [18,19]. Malash [20] indicated that furrow irrigation used 2.6 times more water than drip irrigation, compared to furrow irrigation, drip irrigation increases yield by 11%. Qu [21] studied that compared to flood irrigation, drip irrigation increased crop yield by 6.9-8.2% and 6.5-14.6% over two years, respectively. Zhang [22] studied that drip irrigation under film improves water utilization efficiency by 42.7%, significantly increases peanut yield, and boosts production by 28.8% compared to traditional drip irrigation. Liu [23] showed that compared to border irrigation, drip irrigation increased maize yield by 14.39%, water use efficiency by 43.48-68.09%, and saved about 180 mm of irrigation water.

As is well known, as the soil moisture content increases, the thermal diffusivity gradually increases [24]. While prior studies have extensively explored the relationship between soil moisture and temperature [25–27], the role of irrigation methods in modulating soil thermal regimes remains underexamined, particularly in arid regions like Northwest China. Existing research has primarily focused on short-term (≤2 years) or single-depth measurements [28,29], neglecting the spatiotemporal complexity of soil temperature under varying irrigation regimes. Fail to capture the interannual variations of soil temperature induced by fluctuating meteorological conditions, thereby neglecting the spatiotemporal complexity of soil temperature under varying irrigation regimes. For instance, Zheng [30] demonstrated that CI increased soil surface temperature; however, temperature measurements were limited to the topsoil layers (5 cm and 10 cm), lacking depth-resolved data beyond 20 cm. Similarly, Abdoli [31] emphasized that the topsoil layers (5–15 cm) exhibited the most pronounced temperature fluctuations with significant diurnal variations, yet the study failed to elaborate on the dynamic impacts of different irrigation practices on soil temperature across various soil depths.

This paragraph summarizes the identified research gaps—including the lack of long-term, multi-depth, and multi-positional studies on soil temperature dynamics under different irrigation methods in arid regions of Northwest China, as well as the insufficient understanding of how irrigation-induced soil moisture differences modulate thermal regimes.We conducted a five-year field experiment in Xinjiang. We selected two irrigation methods and three different positions for each irrigation method and proposed the following hypotheses: (1) Under the same soil type and planting mode, whether drip irrigation changes soil temperature. (2) Based on the influence of different irrigation methods on soil moisture distribution characteristics, soil temperature at different positions may have a difference. (3) Under the same irrigation conditions, there are differences in soil temperature at different depths.

## 2. Materials and methods

### 2.1 Experiment site

**Fig 1:**
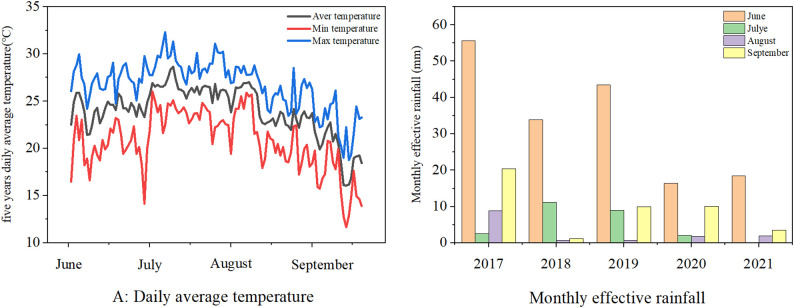
Meteorological variation during the maize growth periods from 2017 to 2021.

This experiment was conducted at the Crop Water Use Experiment Station of the Ministry of Agriculture in Shihezi City, Xinjiang (86°09′E, 45°38′N), from 2017 to 2021. The region has a temperate continental climate and is located in the alluvial fan plain in the northern foothills of the Tianshan Mountains. Meteorological variation during the maize growth periods from 2017 to 2021 is shown in Fig 1. The average temperature during the maize growth season (June-September) was 22.4°C, and the average rainfall was 111.4 mm. Meteorological data were obtained from the Wulanwusu Agrometeorological Experimental Station, located approximately 5 km away from the Crop Water Use Experiment Station of the Ministry of Agriculture in Shihezi City, Xinjiang (86°09′E, 45°38′N). The soil type is grey desert soil and for the topsoil, organic matter content is 7.15 g/kg, total nitrogen 1.41 g/kg, total phosphorus 1.10 g/kg, total potassium 21.59 g/kg, available phosphorus 18.0 mg/kg, available potassium 130.50 mg/kg, average soil bulk density 1.42 g/cm^3^, saturated soil water content (mass water content) was 48.1%, and field capacity was 32% (volumetric water content). The irrigation water source is a deep well; the well is 100 meters deep, and the soil electrical conductivity:0.36–0.54 ds/m (soil-to-water mass ratio 1:5) [32].

### 2.2 Experimental design

The experiment included two irrigation methods: drip irrigation and flood irrigation. The total irrigation volume for both drip irrigation and flood irrigation during the growing season was 4800 m³/ha. The irrigation interval for drip irrigation was 7–9 d, and that for flood irrigation was 12–15 d.The specific irrigation amounts are shown in the Table 1. In the drip irrigation system, a digital flowmeter is installed on the main pipeline connecting the water source and the drip tape to record in real-time the total water volume delivered to each drip-irrigated plot; for flood irrigation, a digital flowmeter is mounted on the inlet pipe of the channel to accurately measure the water volume entering each flood-irrigated plot during each irrigation.

**Table 1:** The specific irrigation amounts.

Treatment		Seeding stage	Jointing stage	Small bell-mouth stage	Big bell-mouth stage	Heading stage	Flowering stage	Silking stage	Grain formation stage	Milk-ripe stage	Total
**DI**	**Irrigation quantity (m**^**3**^ **hm**^**-2**^)	**163.6**	**600.0**	**600.0**	**600.0**	**600.0**	**600.0**	**600.0**	**563.6**	**472.8**	**4800.0**
**MI**		**163.6**	**1200.0**		**1200.0**		**1200.0**		**1036.4**		**4800.0**

**Fig 2:**
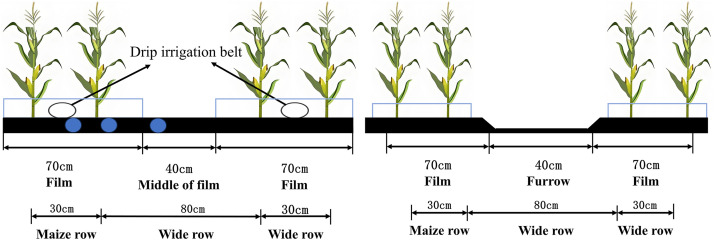
Diagram of drip irrigation and flood irrigation for maize cultivation.

Maize was planted using a planting mode of 1 film, 1 pipe, and 2 rows, with alternating wide (0.8m) and narrow (0.3m) rows. The spacing between maize within a row was 14.4 cm. To maintain uniform seedling emergence across the experiment, all plots were initially irrigated using mulched drip irrigation to promote emergence. After emergence, the plots were divided and field experiments for mulched drip irrigation and flood irrigation were conducted, respectively. Drip irrigation, maize irrigation, sowing, harvesting, and moisture meter installation time are shown in Table 2. The maize cultivation pattern and the location of the moisture meter are shown in Fig 2.

**Table 2:** Maize irrigation, sowing, harvesting, and moisture meter installation time.

Year	Irrigation Method	Irrigation date	Sowing date	Harvest date	Moisture meter installation date
2017	DI	May 7	May 7	September 28	May 5
	MI				
2018	DI	April 28	April 28	September 27	May 12
	MI				
2019	DI	April 30	April 30	September 22	May 10
	MI				
2020	DI	April 26	April 26	October 1	May 9
	MI				
2021	DI	May 7	May 7	September 24	May 19
	MI				

Transparent plastic film for experiments (70 cm wide, 0.01 mm thick) was produced by Xinjiang Tianye Company, Shihezi, China. The drip irrigation belt was selected from the embedded drip irrigation belt produced by Xinjiang Tianye Company, Shihezi, China, with a drip head spacing of 300 mm, a drip head flow rate of 2.0 L/h, and a working pressure of 0.15 MPa. The maize variety used for the experiment was “Zhengdan 958.”

### 2.3 Measurement method

This experiment used the ET-110 Cloud Smart Tubular Soil Moisture Meter (Beijing Dong Fang Run Ze Ecological Technology Co. Ltd.) to measure soil temperature throughout the growth period. A measuring tube was installed at the DS (Dripper Source, 0 cm from the emitter), RZ (Root Zone, 15 cm vertically away from the emitter), and IB (Inter-Row Buffer, 35 cm vertically away from the emitter) of each plot. The measuring depth was 1m, and the vertical spacing between measuring points was 10cm. Automatically monitored soil temperatures at depths of 0-10, 10-20, 20-30, 30-40, 60-70, and 80-90cm, with data being recorded every 10 minutes.

### 2.4 Data analysis

Levene’s test and one-way ANOVA were employed to determine whether significant differences existed across different depths and irrigation methods. Linear regression quantified the relationship between soil and atmospheric temperatures, with model fit evaluated by the coefficient of determination (R²) and significance tested via p-values. All data were statistically analyzed using SPSS 2022, and figures (including soil temperature variations at different sites and depths with error bars) were generated using Origin 2022 and Excel 2017.

## 3. Results

### 3.1. Irrigation methods affect soil temperature during the maize growth periods

The irrigation method significantly affected soil temperature during the maize growing periods (). Based on five-year averaged data from 2017 to 2021, the results demonstrated that drip irrigation (DI) and flood irrigation (MI) exhibited significant differences in soil temperature at 0 cm and 10 cm depths (p < 0.001; p < 0.05, respectively). Under the same soil layer, drip irrigation increased soil temperature by 1.4%. In the 0 cm soil layer, the average temperature across three positions (DS/RZ/IB) under each irrigation method was maintained at approximately 22°C, and the drip irrigation temperature was 22.39°C, which was higher than that of flood irrigation at 22.08°C (p < 0.01). At 10 cm depth, DI showed an average temperature of 21.65°C, 0.12°C higher than MI (p < 0.05). The soil temperature gradient gradually decreased with increasing soil depth. Within the 60-90 cm depth range, both DI and MI maintained stable average temperatures of 20.31°C, exhibiting no significant difference (p > 0.05), indicating diminished influence of irrigation methods on deep soil temperature.

**Fig 3:**
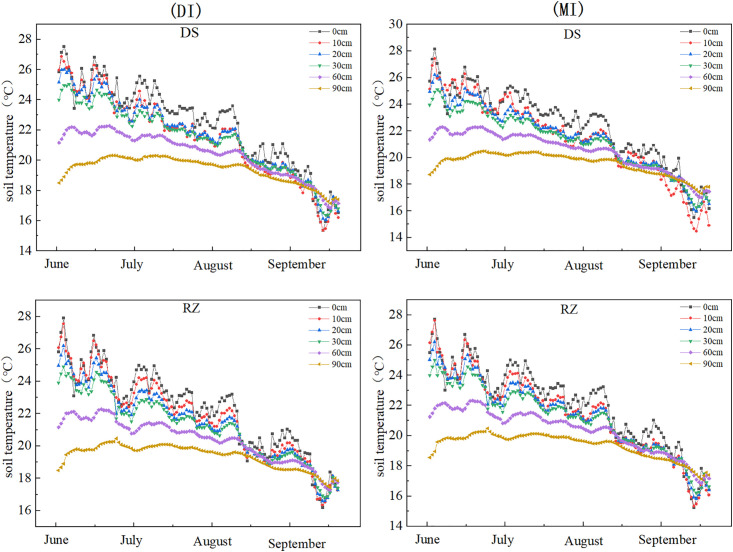
Soil temperatures at the dripper and root zone during the maize growth cycle under different irrigation conditions from 2017 to 2021.

**Fig 4:**
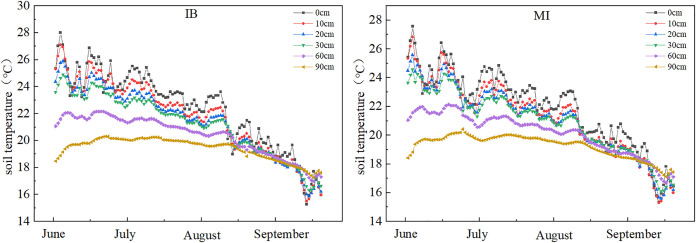
Soil temperatures at the Inter-row Buffer during the maize growth cycle under different irrigation conditions from 2017 to 2021.

**Fig 5:**
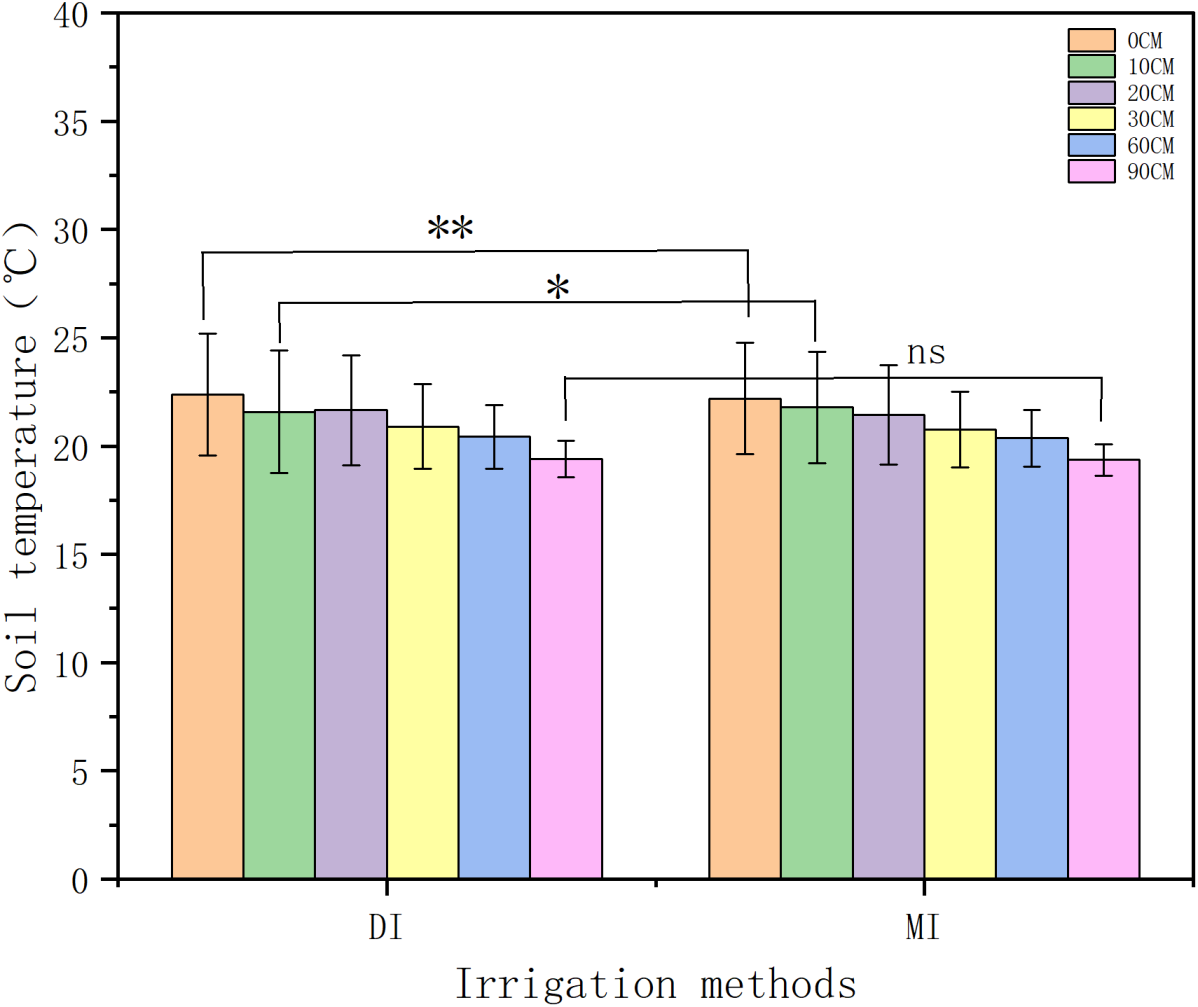
Correlation of soil temperatures at different depths under drip and flood irrigation during the maize growth period from 2017 to 2021. Note: (DI): drip irrigation (MI): Flood irrigation. DS: Dripper Source RZ: Root Zone IB: Inter-row Buffer. *p < 0.05: significant; **p < 0.01/***p < 0.001: extremely significant; ns p > 0.05: no significant difference.

### 3.2. Impact of irrigation methods on soil temperature variations at different depths

Irrigation methods significantly impacted the changes in soil temperature at different depths (). This study, based on the five-year average data from 2017 to 2021, indicates that as the soil depth increases, the soil temperature gradually decreases. The highly significant difference occurs in the surface soil (p<0.001), with the range of changes under drip irrigation being smaller than that under flood irrigation. Specifically, at a depth of 10 cm, the temperature fluctuation range under drip irrigation (DI) (2.32–2.49°C) was significantly smaller than that under flood irrigation (MI) (3.35–3.68°C). indicating superior thermal stability under DI in surface soils. At 40 cm depth, the temperature variation under DI (0.60–0.83°C) was significantly lower than that under MI (1.23–1.35°C), demonstrating that DI reduces temperature fluctuations in deeper soil layers. In the deep soil layer (90 cm), temperatures under both DI and MI remained stable at 19.98–21°C, with no significant difference (p > 0.05). Additionally, flood irrigation resulted in highly significant hysteresis in soil temperature (p < 0.001), with the lowest temperature (20.19°C) observed on 2 days after irrigation, while no such phenomenon occurred under drip irrigation.

**Fig 6:**
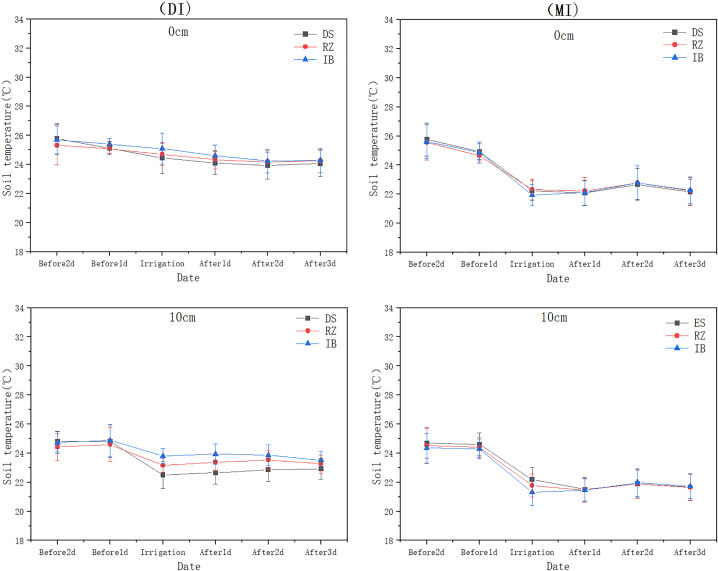
Comparison of soil temperature before and after irrigation with drip and flood irrigation at 0 cm and 10 cm depths.

**Fig 7:**
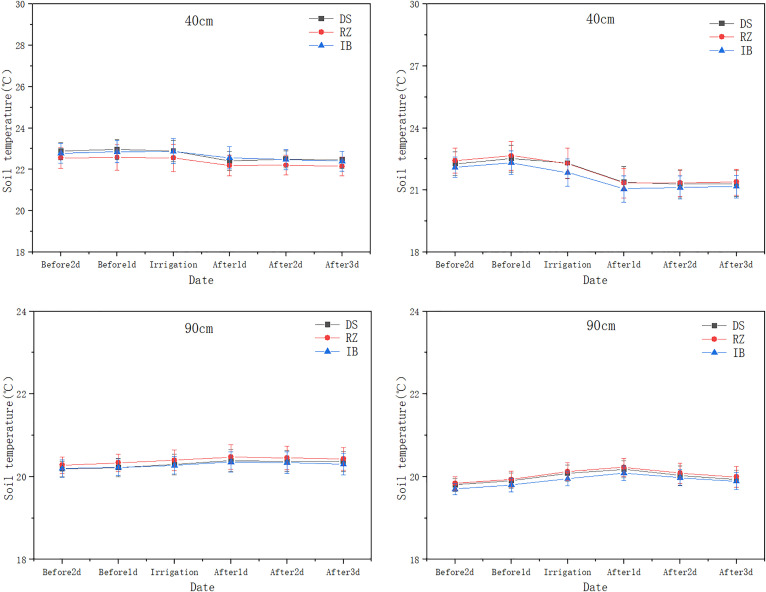
Comparison of soil temperature before and after irrigation with drip and flood irrigation at 40 cm and 90 cm depths.

**Fig 8:**
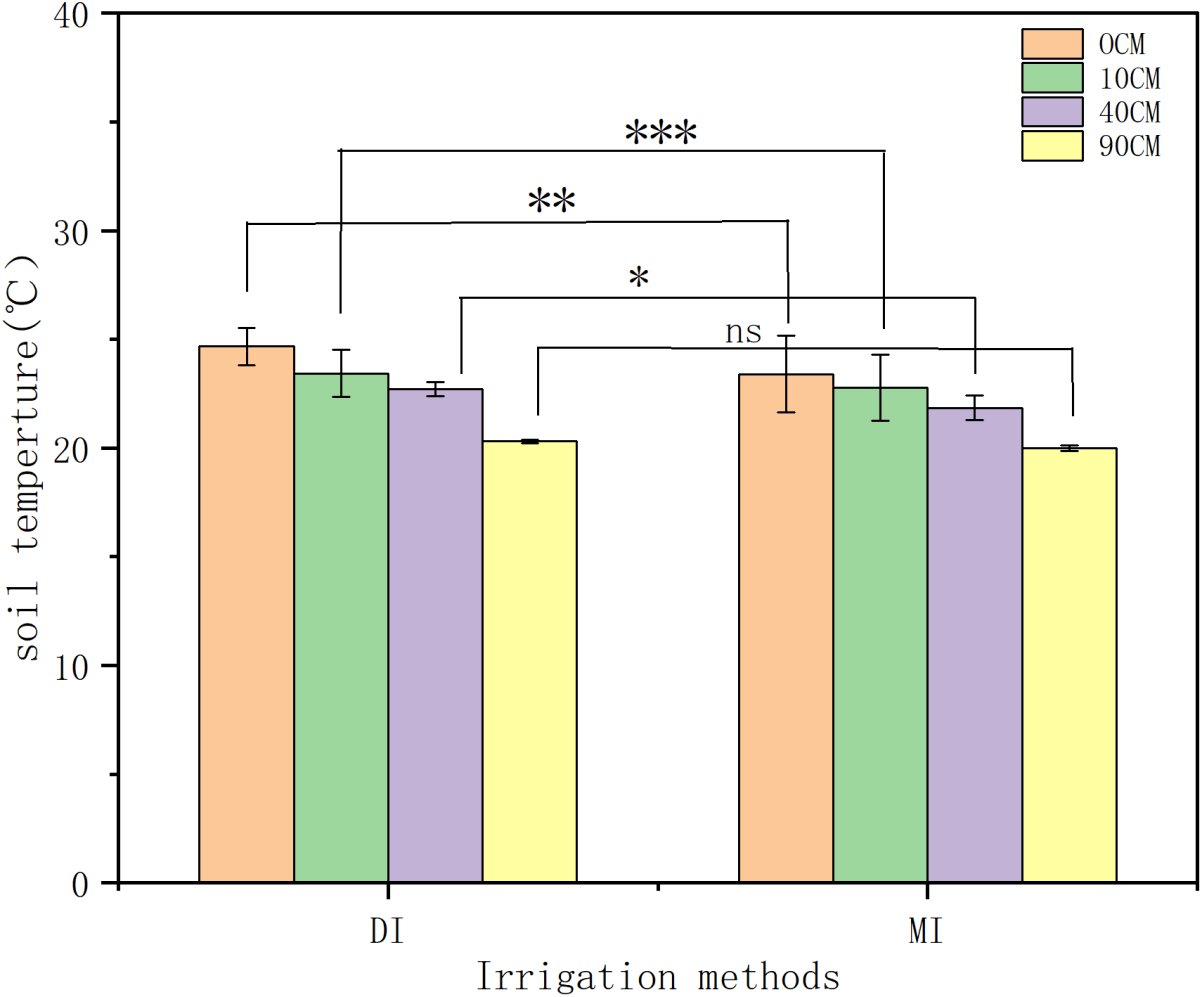
Correlation of soil temperature at different soil depths before and after irrigation under drip and flood Irrigation.

### 3.3. Impact of irrigation methods on daily soil temperature variation

Irrigation methods also have a certain impact on the diurnal temperature amplitude of soil temperature (). The data shown are the average values over the five-year period from 2017 to 2021. Drip irrigation (DI) and flood irrigation (MI) in maximum temperature (16:00) and diurnal temperature amplitude exhibited highly significant differences (p < 0.001). The diurnal temperature amplitude demonstrated significant attenuation with increasing soil depth (p < 0.001). In general, the diurnal temperature ranges at 0 cm depth are 5.79°C -10.55°C, at 10 cm depth are 1.76°C -3.08°C, at 40 cm depth are 0.63°C -1.39°C, and at 90 cm depth are 0.06°C -0.74°C. The variation range at 0 cm depth is approximately 3.61 times that at 10 cm depth, 6.26 times that at 40 cm depth, and 7 times that at 90 cm depth. At depths of 0 cm and 10 cm, the diurnal temperature amplitude in soil temperature is significant; however, both irrigation methods maintained stable temperatures within 0.06–0.74°C at 40–90 cm depths with no significant differences (p > 0.05).

**Fig 9:**
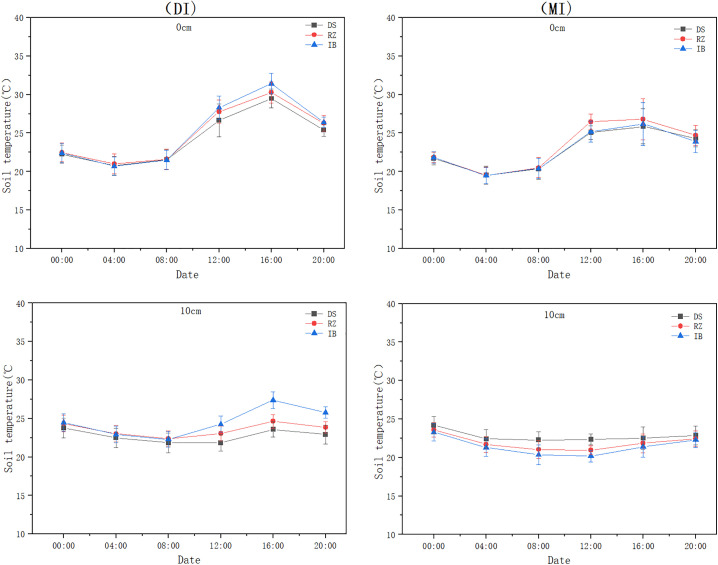
Comparison of diurnal temperature amplitude in soil temperature between drip and flood irrigation at 0 cm and 10 cm depths.

**Fig 10:**
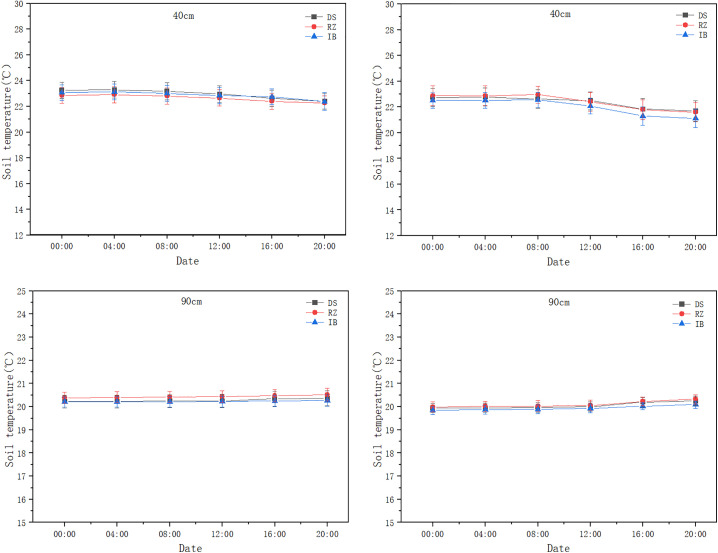
Comparison of diurnal temperature amplitude in soil temperature between drip and flood irrigation at 40 cm and 90 cm depths.

**Fig 11:**
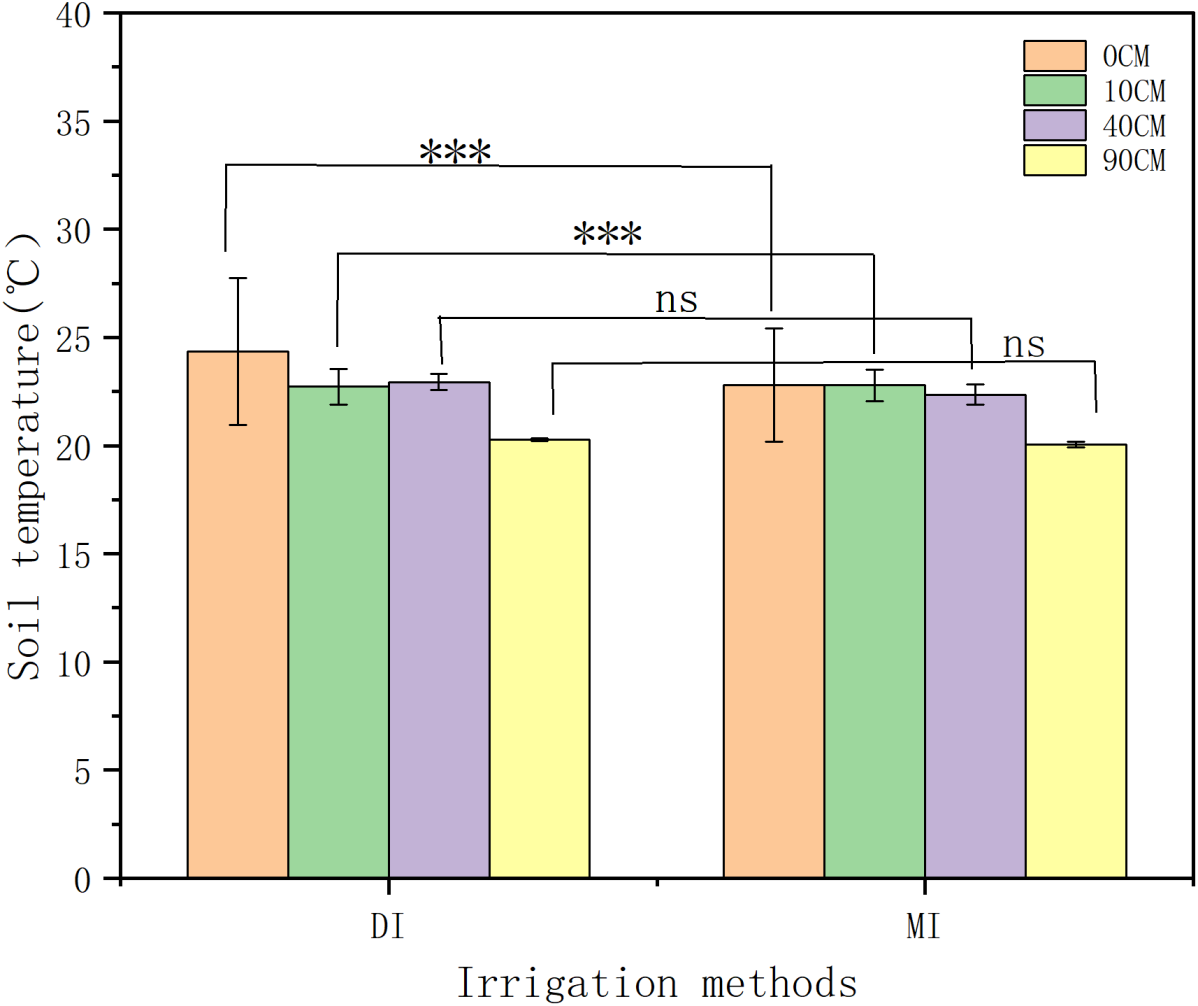
Correlation of soil temperature at different depths and positions in drip and flood irrigation.

### 3.4 Fitting of the relationship between soil temperature and atmospheric temperature at different depths

The soil temperature of six different positions was fitted by a linear equation. The results indicate that the soil surface layer is more susceptible to meteorological influences and positively correlated with meteorological temperature. The results in 5 years (2017-2021) all obtained a good fit. By observing (), we can find that there is an extremely significant correlation between soil temperature and meteorological temperature (p<0.001), effected by irrigation methods and meteorological temperature, the closer R^2^ is to 1, the better the fitting effect. As the soil depth increases, the value of R^2^ gradually decreases. There are differences in R^2^ among different positions, but the changes are not significant. The fitting effect of the soil surface layer is the best, and the fitting effect of drip irrigation is more significant compared to flood irrigation (the average value of R^2^ under drip irrigation is 0.827; R^2^ under flood irrigation is 0.738).

**Fig 12:**
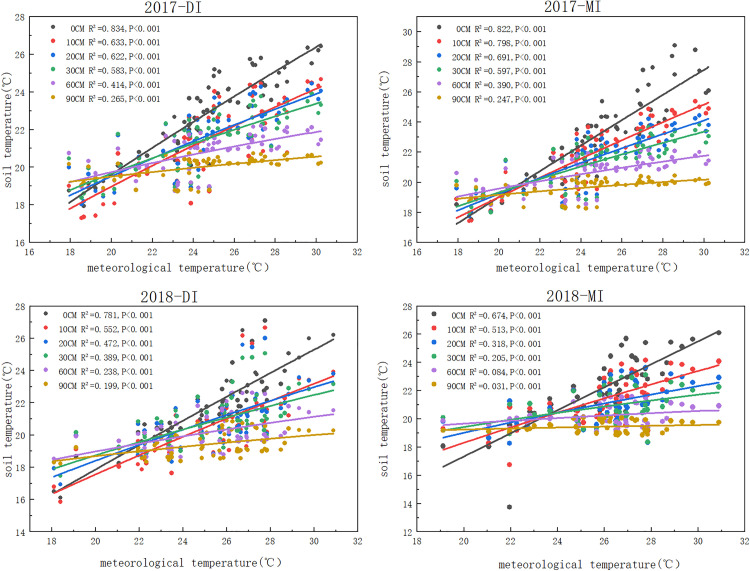
Linear fitting relationship between soil temperature and Meteorological Temperature under Drip Irrigation and Flood Irrigation in 2017 and 2018.

**Fig 13:**
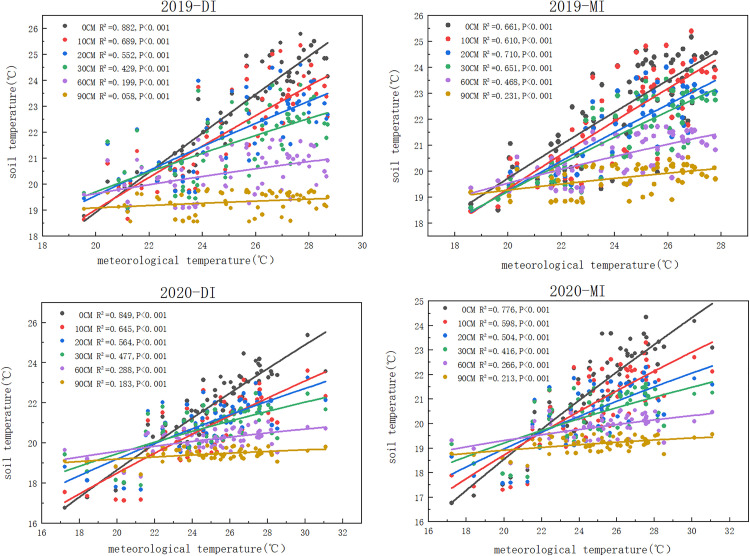
Linear Fitting Relationship between Soil Temperature and Meteorological Temperature under Drip Irrigation and Flood Irrigation in 2019 and 2020.

**Fig 14:**
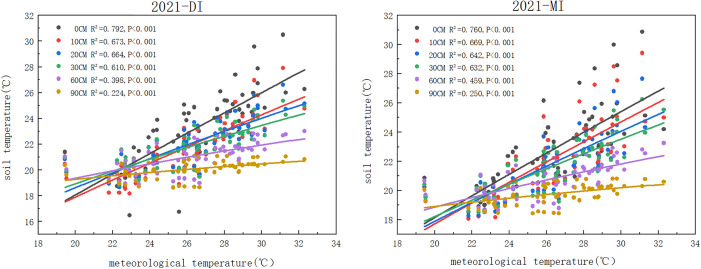
Linear Fitting Relationship between Soil Temperature and Meteorological Temperature under Drip Irrigation and Flood Irrigation in 2021.

## 4. Discussion

### 4.1 Irrigation methods affect soil temperature during the maize growth periods

Drip irrigation differs from flood irrigation in that it has a shorter irrigation cycle and a smaller irrigation volume. Drip irrigation increased soil temperature by 1.4% compared to flood irrigation, primarily due to reduced evaporation and improved heat retention under plastic mulching [33,34]. Biswal [35] highlighted that limited drip irrigation resulted in a soil temperature increase of 2°C when compared to furrow irrigation. Yang [36] revealed that mulched drip irrigation, compared to traditional irrigation, led to an increase in soil moisture content by 13.6-25% and soil temperature by 2-4°C in the 10-50 cm soil layer. Li [37] found that soil temperatures in the 0-25 cm soil layer under mulching conditions increased by 2.29-4.61% compared to bare ground irrigation. Consistent with previous studies, plastic film mulching reduces soil evaporation and enhances soil temperature retention [33,38], which is similar to the findings of this study. In addition, drip irrigation under the membrane changed the irrigation period, irrigation frequency, and irrigation quota of traditional irrigation [39,40]. Changes in irrigation frequency resulted in significant changes in soil water content [41]. There is an obvious interaction between soil moisture content and temperature [13]. Liu [42] showed that as the soil moisture content increases, the thermal diffusivity gradually increases, but it decreases with further increases in moisture content. Therefore, drip irrigation will not only reduce soil evaporation but also be more beneficial for soil warming. However, this warming effect may accelerate soil organic matter (SOM) decomposition through increased microbial activity [43]. Previous studies have shown that each 1°C increase in temperature elevates soil respiration rates by approximately 8–10%, resulting in an annual SOC (soil organic carbon) loss of 0.5–1.5% [44]. Although elevated soil temperatures enhance crop productivity, prolonged drip irrigation in Xinjiang’s gray desert soils may accelerate microbial activity, intensifying organic carbon decomposition and thereby threatening long-term soil productivity sustainability.

### 4.2 Irrigation methods affect soils temperature at different depths

Different irrigation methods lead to different infiltration patterns and depths of infiltration. The irrigation water falls on the soil surface in drops or thin streams, concentrating moisture near the surface. This study showed a notable fluctuation in the surface soil temperature at 0 cm, ranging from 19.79°C to 28.38°C, which gradually stabilized as the soil depth increased. Zarei [45] showed that at a depth of 10 cm, compared to surface irrigation, wick irrigation significantly increased the soil moisture content by 50%, 70%, and 100%. However, with increasing soil depth, temperature fluctuations in irrigation systems decreased. Xiao [46] observed that alterations in irrigation volume led to a temperature increase of 2.8%-7.06% and 2.69%-8.74%, with the most significant differences occurring at the shallow soil depths of 5 cm and 10 cm. The results of our study are similar to these studies. Wang [47] highlighted that soil moisture and temperature transport processes are highly similar, as the soil depth increases, the magnitude of change decreases. The moisture in the soil has a high specific heat capacity and high thermal conductivity, resulting in relatively slower soil heating and cooling rates. Therefore, at a certain soil moisture content, temperature fluctuations in the soil may be relatively small [48].

Besides, external factors such as atmospheric temperature may also affect the surface soil temperature [49]. Soil temperature is positively correlated with atmospheric temperature. Qiao [50] showed that the temperature of the soil surface is mainly influenced by the intensity of solar radiation. Li [51] showed that surface soil temperature was maintained within the range of 19°C to 31°C in response to ambient temperatures, while temperature changes in the soil layer between 40-60 cm exhibited a slower rate of change. The findings of García [52] and others similarly demonstrate this trend.

### 4.3 Irrigation methods significantly affect soil temperature at maize root zone

Different irrigation methods have different water supply patterns, which to a certain extent affect the distribution of water in the soil [18]. This study showed that under drip irrigation conditions, the soil temperature in the root zone increased by 0.5% to 2.25% compared to other positions. Drip irrigation preferentially replenishes the water near the dripper source and soil root zone, while flood irrigation preferentially replenishes the soil moisture in the wide rows [53]. Drip irrigation supplies a little amount of water to maize roots through high-frequency watering, enabling the maize to maintain optimal water conditions and forming an ellipsoidal or spherical wetting zone in the maize root zone, thereby effectively reducing deep infiltration [54]. Drip irrigation is a more targeted irrigation method than flood irrigation, with soil moisture concentrated mostly in the maize root zone [55]. Cheng [56] showed that drip irrigation enhanced the soil moisture content of both seedlings and maize roots by 59-207% and 72-173%, respectively, compared to boundary irrigation. As widely recognized, changes in soil moisture content influence soil thermal conductivity more than any other factor [57], water has a relatively high specific heat capacity [58]. Consequently, drip irrigation optimizes heat absorption and storage in the rhizosphere, further promoting maize growth and development [59], enabling it to maintain suitable temperature and humidity conditions, which in turn creates favorable conditions for maize growth. However, this research was mainly carried out in Xinjiang, focusing on grey desert soil. Therefore, the data may differ from those of other regions.

This study primarily focused on gray desert soils in Xinjiang; the data may differ in soils with distinct textures (clay or loam). The semi-arid climate of Xinjiang limits the applicability of the findings to regions with divergent precipitation patterns (e.g., monsoon-affected areas). Despite these limitations, the findings demonstrate the temperature-regulating advantages of drip irrigation in arid northwestern China. Future research could focus on the following directions: (1) Prioritize integrated nutrient management to mitigate potential SOM loss. (2) Conduct site-specific trials to optimize irrigation schedules for different soil types. (3) Monitor long-term soil health indicators under drip irrigation systems.

## 5. Conclusions

This study conducted a five-year field experiment in the northwestern arid region of China, significantly advancing the mechanistic understanding of spatiotemporal soil temperature dynamics under water-saving drip irrigation compared to conventional flood irrigation. We established a long-term and multi-dimensional monitoring system, filling the gap in understanding irrigation-induced soil thermal regime changes in arid regions where short-term and single-depth studies dominate. For the first time, we demonstrated that drip irrigation preferentially increases soil temperature in the root zone (0.5–2.25% higher than other positions), providing direct evidence of its optimization of hydrothermal conditions for crop roots. Specifically, drip irrigation increased soil temperature during the maize growth periods, the soil surface layer is more susceptible to meteorological influences and positively correlated with atmospheric temperature. The surface soil temperature (0-10cm) changed more dramatically than that of deeper layers, and the soil temperature near the maize root zone is slightly higher than that of other positions. Under drip irrigation conditions, the soil temperature was 0.31°C higher than flood irrigation, increasing by 1.4%. The soil temperature in the root zone increased by 0.5% to 2.25% compared to other positions. Under the same soil texture and planting method, drip irrigation shows significant advantages in increasing soil temperature, especially in the maize root zone.

Thus, this five-year field experiment confirms that drip irrigation not only increases soil temperature by 1.4% compared to flood irrigation but also preferentially raises root zone temperature by 0.5%–2.25%, optimizing hydrothermal conditions for maize roots and providing a scientific foundation for sustainable agricultural water management in arid northwestern China.
